# 2-[2-(Hydroxy­meth­yl)phen­yl]-1-(1-naphth­yl)ethanol

**DOI:** 10.1107/S1600536810000383

**Published:** 2010-01-16

**Authors:** F. Nawaz Khan, P. Manivel, Venkatesha R. Hathwar, V. Krishnakumar, Richa Tyagi

**Affiliations:** a Chemistry Division, School of Advanced Science, VIT University, Vellore 632 014, Tamil Nadu, India; b Solid State and Structural Chemistry Unit, Indian Institute of Science, Bangalore 560 012, Karnataka, India; c Chemistry Department, Hindu College, Delhi University, Delhi 110 007, India

## Abstract

The mol­ecular conformation of the title compound, C_19_H_18_O_2_, is stabilized by an intra­molecular O—H—O hydrogen bond. In addition, inter­molecular O—H—O inter­actions link the mol­ecules into zigzag chains running along the *c* axis.

## Related literature

For related structures, see: Gałdecki *et al.* (1984 ▶); Hoyos-Guerrero *et al.* (1983 ▶); Manivel *et al.* (2009 ▶).
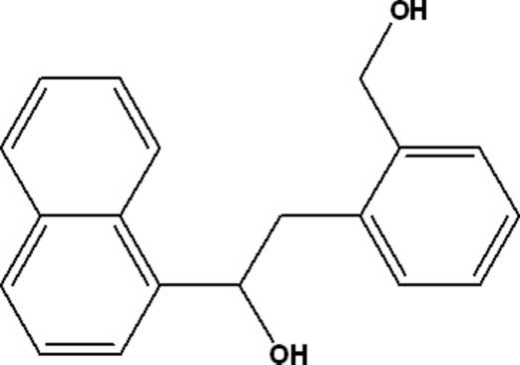


## Experimental

### 

#### Crystal data

C_19_H_18_O_2_
*M* *_r_* = 278.33
Monoclinic, 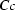

*a* = 16.207 (4) Å
*b* = 12.820 (3) Å
*c* = 7.7888 (18) Å
β = 111.172 (3)°
*V* = 1509.2 (6) Å^3^
*Z* = 4
Mo *K*α radiation
μ = 0.08 mm^−1^
*T* = 290 K
0.60 × 0.10 × 0.10 mm

#### Data collection

Bruker SMART CCD area-detector diffractometer
Absorption correction: multi-scan (*SADABS*; Sheldrick, 1996 ▶) *T*_min_ = 0.943, *T*_max_ = 0.992
5447 measured reflections
1447 independent reflections
1216 reflections with *I* > 2σ(*I*)
*R*_int_ = 0.039

#### Refinement

*R*[*F* ^2^ > 2σ(*F* ^2^)] = 0.038
*wR*(*F* ^2^) = 0.085
*S* = 1.07
1447 reflections
198 parameters
2 restraints
H atoms treated by a mixture of independent and constrained refinement
Δρ_max_ = 0.15 e Å^−3^
Δρ_min_ = −0.14 e Å^−3^

### 

Data collection: *SMART* (Bruker, 2004 ▶); cell refinement: *SAINT* (Bruker, 2004 ▶); data reduction: *SAINT*; program(s) used to solve structure: *SHELXS97* (Sheldrick, 2008 ▶); program(s) used to refine structure: *SHELXL97* (Sheldrick, 2008 ▶); molecular graphics: *ORTEP-3* (Farrugia, 1997 ▶) and *CAMERON* (Watkin *et al.*, 1993 ▶); software used to prepare material for publication: *PLATON* (Spek, 2009 ▶).

## Supplementary Material

Crystal structure: contains datablocks global, I. DOI: 10.1107/S1600536810000383/bt5147sup1.cif

Structure factors: contains datablocks I. DOI: 10.1107/S1600536810000383/bt5147Isup2.hkl

Additional supplementary materials:  crystallographic information; 3D view; checkCIF report

## Figures and Tables

**Table 1:** Hydrogen-bond geometry (Å, °)

*D*—H⋯*A*	*D*—H	H⋯*A*	*D*⋯*A*	*D*—H⋯*A*
O1—H1*O*⋯O2^i^	0.87 (4)	1.94 (4)	2.721 (3)	148 (4)
O2—H2*O*⋯O1	0.94 (5)	1.79 (4)	2.721 (3)	169 (4)

Symmetry code: (i) 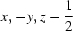.

**Fig. 1.**
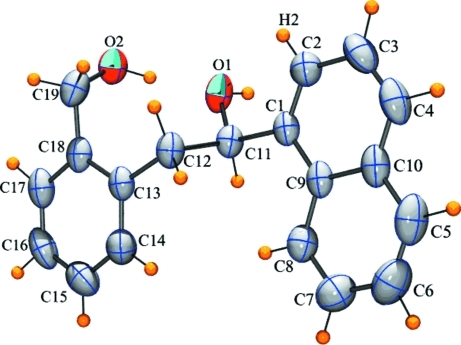
ORTEP diagram of the title compound with 50% probability displacement ellipsoids.

**Fig. 2.**
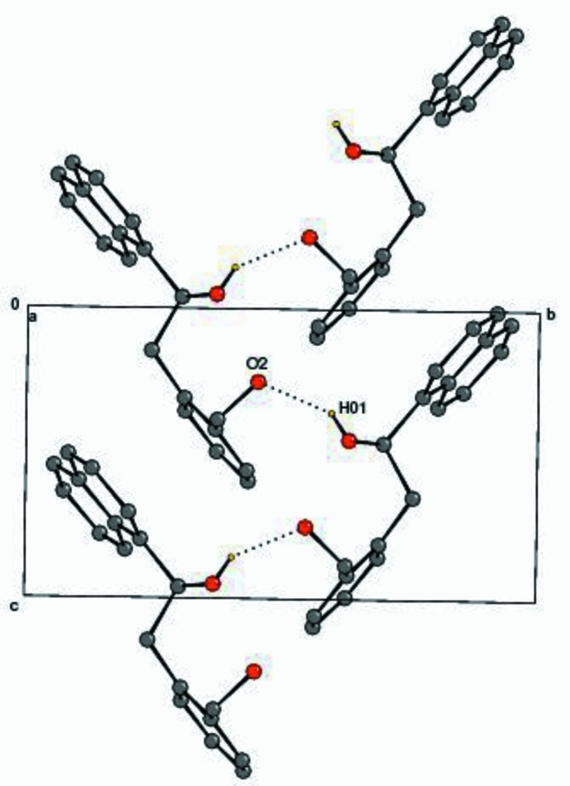
The crystal packing diagram. The dotted lines indicate intermolecular O—H···O hydrogen bonds.

## supplementary crystallographic information

### Comment

The molecular conformation of the  title  compound is stabilized by an intramolecular O—H—O hydrogen bond. In addition,   intermolecular O—H—O interactions link the molecules to zigzag chains running along the c axis.

### Experimental

3-(naphthalen-1-yl)isocoumarin (1 eq.) was dissolved in 10 volumes of methanol, sodium borohydride (4 eq.) was added to it and stirred at 50° C under nitrogen atmosphere for 4 hrs. Then two more equivalents of NaBH_4_ was further added and left overnight at 50° C for completion of the reaction. After TLC analysis, solvent methanol was removed, extracted with ethyl acetate. The ethyl acetate layer was washed with water, dried with anhydrous Na_2_SO_4_, evaporated to yield the title compound, which was further purified by washing with petroleum ether. Single-crystals for the structure analysis were obtained by slow evaporation of the ethanol solution.

### Refinement

In the absence of anomalous scatterers, 1191 Friedel pairs were merged and the absolute configuration was arbitrarily set. All   H atoms were located from difference fourier maps Those bonded to C  were positioned geometrically and refined using a riding model with C—H bond lengths of 0.93 Å and 0.97 Å for aromatic and for methylene H atoms, respectively, and *U*_iso_(H) = 1.2*U*_eq_(C). The  hydroxyl H atoms were freely refined.

### Crystal data

**Table:**

C_19_H_18_O_2_	*F*(000) = 592
*M**_r_* = 278.33	*D*_x_ = 1.225 Mg m^−^^3^
Monoclinic, *C**c*	Mo *K*α radiation, λ = 0.71073 Å
Hall symbol: C -2yc	Cell parameters from 2097 reflections
*a* = 16.207 (4) Å	θ = 2.7–26.3°
*b* = 12.820 (3) Å	µ = 0.08 mm^−^^1^
*c* = 7.7888 (18) Å	*T* = 290 K
β = 111.172 (3)°	Needle, colorless
*V* = 1509.2 (6) Å^3^	0.60 × 0.10 × 0.10 mm
*Z* = 4	

### Data collection

**Table:**

Bruker SMART CCD area-detector diffractometer	1447 independent reflections
Radiation source: fine-focus sealed tube	1216 reflections with *I* > 2σ(*I*)
graphite	*R*_int_ = 0.039
φ and ω scans	θ_max_ = 25.7°, θ_min_ = 2.1°
Absorption correction: multi-scan (*SADABS*; Sheldrick, 1996)	*h* = −19→19
*T*_min_ = 0.943, *T*_max_ = 0.992	*k* = −15→15
5447 measured reflections	*l* = −9→9

### Refinement

**Table:**

Refinement on *F*^2^	Primary atom site location: structure-invariant direct methods
Least-squares matrix: full	Secondary atom site location: difference Fourier map
*R*[*F*^2^ > 2σ(*F*^2^)] = 0.038	Hydrogen site location: inferred from neighbouring sites
*wR*(*F*^2^) = 0.085	H atoms treated by a mixture of independent and constrained refinement
*S* = 1.07	*w* = 1/[σ^2^(*F*_o_^2^) + (0.0524*P*)^2^] where *P* = (*F*_o_^2^ + 2*F*_c_^2^)/3
1447 reflections	(Δ/σ)_max_ < 0.001
198 parameters	Δρ_max_ = 0.15 e Å^−^^3^
2 restraints	Δρ_min_ = −0.14 e Å^−^^3^

### Special details

**Table:**

Geometry. All s.u.'s (except the s.u. in the dihedral angle between two l.s. planes) are estimated using the full covariance matrix. The cell s.u.'s are taken into account individually in the estimation of s.u.'s in distances, angles and torsion angles; correlations between s.u.'s in cell parameters are only used when they are defined by crystal symmetry. An approximate (isotropic) treatment of cell s.u.'s is used for estimating s.u.'s involving l.s. planes.
Refinement. Refinement of *F*^2^ against ALL reflections. The weighted *R*-factor *wR* and goodness of fit *S* are based on *F*^2^, conventional *R*-factors *R* are based on *F*, with *F* set to zero for negative *F*^2^. The threshold expression of *F*^2^ > 2σ(*F*^2^) is used only for calculating *R*-factors(gt) *etc*. and is not relevant to the choice of reflections for refinement. *R*-factors based on *F*^2^ are statistically about twice as large as those based on *F*, and *R*- factors based on ALL data will be even larger.

### Fractional atomic coordinates and isotropic or equivalent isotropic displacement parameters (Å^2^)

**Table:**

	*x*	*y*	*z*	*U*_iso_*/*U*_eq_	
O1	0.07843 (13)	0.13119 (13)	0.4503 (3)	0.0541 (5)	
H1O	0.074 (2)	0.091 (3)	0.357 (6)	0.100 (13)*	
O2	0.05537 (13)	0.04804 (13)	0.7513 (3)	0.0541 (5)	
H2O	0.068 (3)	0.070 (3)	0.647 (6)	0.112 (14)*	
C1	0.12467 (16)	0.27456 (16)	0.2976 (3)	0.0391 (5)	
C2	0.03809 (18)	0.2984 (2)	0.2052 (4)	0.0513 (6)	
H2	−0.0052	0.2669	0.2399	0.062*	
C3	0.0124 (2)	0.3708 (2)	0.0562 (4)	0.0633 (8)	
H3	−0.0472	0.3859	−0.0056	0.076*	
C4	0.0748 (2)	0.4177 (2)	0.0047 (4)	0.0609 (8)	
H4	0.0575	0.4648	−0.0926	0.073*	
C5	0.2323 (3)	0.4454 (2)	0.0452 (4)	0.0681 (9)	
H5	0.2157	0.4922	−0.0527	0.082*	
C6	0.3193 (3)	0.4256 (2)	0.1359 (5)	0.0760 (9)	
H6	0.3617	0.4597	0.1020	0.091*	
C7	0.3451 (2)	0.3536 (2)	0.2810 (5)	0.0672 (8)	
H7	0.4049	0.3393	0.3419	0.081*	
C8	0.28404 (18)	0.30420 (19)	0.3341 (4)	0.0514 (7)	
H8	0.3029	0.2566	0.4305	0.062*	
C9	0.19190 (16)	0.32361 (16)	0.2457 (3)	0.0405 (5)	
C10	0.16515 (18)	0.39629 (17)	0.0958 (3)	0.0476 (6)	
C11	0.15080 (17)	0.19895 (15)	0.4609 (3)	0.0414 (5)	
H11	0.2007	0.1563	0.4591	0.050*	
C12	0.17832 (17)	0.25725 (16)	0.6467 (3)	0.0445 (6)	
H12A	0.2115	0.3191	0.6400	0.053*	
H12B	0.1254	0.2799	0.6668	0.053*	
C13	0.23412 (17)	0.19156 (17)	0.8103 (3)	0.0415 (5)	
C14	0.32550 (19)	0.1898 (2)	0.8541 (4)	0.0556 (7)	
H14	0.3500	0.2320	0.7877	0.067*	
C15	0.3809 (2)	0.1274 (2)	0.9933 (4)	0.0640 (8)	
H15	0.4415	0.1270	1.0184	0.077*	
C16	0.3454 (2)	0.0655 (2)	1.0947 (4)	0.0625 (8)	
H16	0.3818	0.0223	1.1868	0.075*	
C17	0.2558 (2)	0.06847 (18)	1.0582 (3)	0.0551 (7)	
H17	0.2325	0.0283	1.1292	0.066*	
C18	0.19887 (17)	0.13022 (15)	0.9173 (3)	0.0430 (6)	
C19	0.1015 (2)	0.1264 (2)	0.8835 (4)	0.0537 (7)	
H19A	0.0936	0.1123	0.9990	0.064*	
H19B	0.0756	0.1940	0.8399	0.064*	

### Atomic displacement parameters (Å^2^)

**Table:**

	*U*^11^	*U*^22^	*U*^33^	*U*^12^	*U*^13^	*U*^23^
O1	0.0811 (13)	0.0413 (9)	0.0459 (10)	−0.0112 (9)	0.0300 (9)	−0.0034 (8)
O2	0.0705 (11)	0.0446 (9)	0.0504 (10)	−0.0041 (8)	0.0259 (9)	0.0052 (8)
C1	0.0571 (15)	0.0258 (10)	0.0338 (12)	0.0073 (10)	0.0156 (11)	−0.0023 (10)
C2	0.0587 (17)	0.0462 (14)	0.0491 (15)	0.0068 (12)	0.0194 (13)	−0.0007 (12)
C3	0.0707 (18)	0.0563 (16)	0.0486 (16)	0.0238 (15)	0.0042 (14)	0.0046 (14)
C4	0.093 (2)	0.0376 (13)	0.0428 (15)	0.0156 (15)	0.0134 (15)	0.0089 (12)
C5	0.119 (3)	0.0344 (14)	0.0600 (18)	−0.0026 (16)	0.0431 (19)	0.0079 (13)
C6	0.098 (3)	0.0610 (19)	0.084 (2)	−0.0149 (18)	0.051 (2)	0.0039 (18)
C7	0.0674 (19)	0.0587 (18)	0.081 (2)	−0.0007 (14)	0.0338 (17)	0.0029 (16)
C8	0.0678 (18)	0.0395 (13)	0.0498 (16)	0.0080 (12)	0.0248 (14)	0.0057 (12)
C9	0.0617 (16)	0.0258 (10)	0.0339 (12)	0.0059 (10)	0.0172 (11)	−0.0024 (9)
C10	0.0792 (19)	0.0254 (10)	0.0388 (14)	0.0043 (11)	0.0220 (14)	−0.0010 (10)
C11	0.0572 (14)	0.0286 (10)	0.0410 (13)	0.0049 (11)	0.0210 (11)	0.0027 (10)
C12	0.0638 (17)	0.0292 (11)	0.0421 (13)	−0.0002 (11)	0.0211 (12)	0.0019 (10)
C13	0.0595 (17)	0.0294 (11)	0.0350 (12)	−0.0003 (10)	0.0162 (11)	−0.0034 (9)
C14	0.0640 (19)	0.0554 (16)	0.0471 (15)	−0.0051 (14)	0.0197 (13)	−0.0004 (13)
C15	0.0588 (17)	0.0668 (18)	0.0540 (18)	0.0036 (14)	0.0055 (14)	−0.0052 (15)
C16	0.083 (2)	0.0468 (15)	0.0401 (15)	0.0099 (14)	0.0012 (14)	0.0023 (12)
C17	0.091 (2)	0.0349 (13)	0.0390 (14)	−0.0010 (13)	0.0228 (14)	−0.0008 (11)
C18	0.0658 (17)	0.0276 (10)	0.0372 (13)	0.0008 (10)	0.0205 (12)	−0.0053 (10)
C19	0.078 (2)	0.0406 (14)	0.0529 (16)	0.0035 (12)	0.0364 (14)	0.0016 (12)

### Geometric parameters (Å, °)

**Table:**

O1—C11	1.438 (3)	C8—H8	0.9300
O1—H1O	0.87 (4)	C9—C10	1.433 (3)
O2—C19	1.439 (3)	C11—C12	1.544 (3)
O2—H2O	0.94 (5)	C11—H11	0.9800
C1—C2	1.361 (3)	C12—C13	1.523 (3)
C1—C9	1.437 (3)	C12—H12A	0.9700
C1—C11	1.533 (3)	C12—H12B	0.9700
C2—C3	1.425 (4)	C13—C14	1.395 (4)
C2—H2	0.9300	C13—C18	1.408 (3)
C3—C4	1.356 (5)	C14—C15	1.386 (4)
C3—H3	0.9300	C14—H14	0.9300
C4—C10	1.404 (4)	C15—C16	1.382 (4)
C4—H4	0.9300	C15—H15	0.9300
C5—C6	1.353 (5)	C16—C17	1.375 (5)
C5—C10	1.430 (4)	C16—H16	0.9300
C5—H5	0.9300	C17—C18	1.397 (3)
C6—C7	1.402 (5)	C17—H17	0.9300
C6—H6	0.9300	C18—C19	1.504 (4)
C7—C8	1.358 (4)	C19—H19A	0.9700
C7—H7	0.9300	C19—H19B	0.9700
C8—C9	1.422 (4)		
			
C11—O1—H1O	103 (3)	C1—C11—C12	111.80 (16)
C19—O2—H2O	101 (3)	O1—C11—H11	108.8
C2—C1—C9	119.7 (2)	C1—C11—H11	108.8
C2—C1—C11	120.3 (2)	C12—C11—H11	108.8
C9—C1—C11	120.0 (2)	C13—C12—C11	113.55 (17)
C1—C2—C3	121.3 (3)	C13—C12—H12A	108.9
C1—C2—H2	119.4	C11—C12—H12A	108.9
C3—C2—H2	119.4	C13—C12—H12B	108.9
C4—C3—C2	120.0 (3)	C11—C12—H12B	108.9
C4—C3—H3	120.0	H12A—C12—H12B	107.7
C2—C3—H3	120.0	C14—C13—C18	117.9 (2)
C3—C4—C10	121.1 (2)	C14—C13—C12	118.1 (2)
C3—C4—H4	119.4	C18—C13—C12	124.0 (2)
C10—C4—H4	119.4	C15—C14—C13	122.1 (3)
C6—C5—C10	121.8 (3)	C15—C14—H14	119.0
C6—C5—H5	119.1	C13—C14—H14	119.0
C10—C5—H5	119.1	C16—C15—C14	119.5 (3)
C5—C6—C7	119.7 (3)	C16—C15—H15	120.2
C5—C6—H6	120.2	C14—C15—H15	120.2
C7—C6—H6	120.2	C17—C16—C15	119.5 (3)
C8—C7—C6	120.9 (3)	C17—C16—H16	120.3
C8—C7—H7	119.5	C15—C16—H16	120.3
C6—C7—H7	119.5	C16—C17—C18	121.8 (2)
C7—C8—C9	121.6 (2)	C16—C17—H17	119.1
C7—C8—H8	119.2	C18—C17—H17	119.1
C9—C8—H8	119.2	C17—C18—C13	119.2 (2)
C8—C9—C10	117.6 (2)	C17—C18—C19	118.2 (2)
C8—C9—C1	123.9 (2)	C13—C18—C19	122.6 (2)
C10—C9—C1	118.5 (2)	O2—C19—C18	112.9 (2)
C4—C10—C5	122.2 (2)	O2—C19—H19A	109.0
C4—C10—C9	119.5 (2)	C18—C19—H19A	109.0
C5—C10—C9	118.3 (3)	O2—C19—H19B	109.0
O1—C11—C1	111.1 (2)	C18—C19—H19B	109.0
O1—C11—C12	107.45 (19)	H19A—C19—H19B	107.8
			
C9—C1—C2—C3	0.5 (3)	C2—C1—C11—O1	23.3 (3)
C11—C1—C2—C3	178.2 (2)	C9—C1—C11—O1	−159.00 (19)
C1—C2—C3—C4	−0.3 (4)	C2—C1—C11—C12	−96.7 (3)
C2—C3—C4—C10	0.0 (4)	C9—C1—C11—C12	81.0 (2)
C10—C5—C6—C7	1.4 (5)	O1—C11—C12—C13	78.1 (2)
C5—C6—C7—C8	−1.0 (5)	C1—C11—C12—C13	−159.79 (19)
C6—C7—C8—C9	−0.2 (4)	C11—C12—C13—C14	86.8 (3)
C7—C8—C9—C10	0.9 (3)	C11—C12—C13—C18	−91.5 (3)
C7—C8—C9—C1	−179.2 (3)	C18—C13—C14—C15	2.4 (3)
C2—C1—C9—C8	179.7 (2)	C12—C13—C14—C15	−176.0 (2)
C11—C1—C9—C8	2.1 (3)	C13—C14—C15—C16	−0.9 (4)
C2—C1—C9—C10	−0.4 (3)	C14—C15—C16—C17	−1.2 (4)
C11—C1—C9—C10	−178.10 (18)	C15—C16—C17—C18	1.9 (4)
C3—C4—C10—C5	−179.4 (3)	C16—C17—C18—C13	−0.3 (3)
C3—C4—C10—C9	0.1 (4)	C16—C17—C18—C19	178.3 (2)
C6—C5—C10—C4	178.8 (3)	C14—C13—C18—C17	−1.8 (3)
C6—C5—C10—C9	−0.7 (4)	C12—C13—C18—C17	176.5 (2)
C8—C9—C10—C4	180.0 (2)	C14—C13—C18—C19	179.7 (2)
C1—C9—C10—C4	0.2 (3)	C12—C13—C18—C19	−2.0 (3)
C8—C9—C10—C5	−0.5 (3)	C17—C18—C19—O2	−90.6 (3)
C1—C9—C10—C5	179.7 (2)	C13—C18—C19—O2	88.0 (3)

### Hydrogen-bond geometry (Å, °)

**Table:**

*D*—H···*A*	*D*—H	H···*A*	*D*···*A*	*D*—H···*A*
O1—H1O···O2^i^	0.87 (4)	1.94 (4)	2.721 (3)	148 (4)
O2—H2O···O1	0.94 (5)	1.79 (4)	2.721 (3)	169 (4)

Symmetry codes: (i) *x*, −*y*, *z*−1/2.
